# CYP98A22, a phenolic ester 3’-hydroxylase specialized in the synthesis of chlorogenic acid, as a new tool for enhancing the furanocoumarin concentration in *Ruta graveolens*

**DOI:** 10.1186/1471-2229-12-152

**Published:** 2012-08-29

**Authors:** Fazeelat Karamat, Alexandre Olry, Sébastien Doerper, Guilhem Vialart, Pascaline Ullmann, Danièle Werck-Reichhart, Frédéric Bourgaud, Alain Hehn

**Affiliations:** 1 Université de Lorraine UMR 1121, Agronomie et Environnement Nancy-Colmar, ENSAIA 2 avenue de la forêt de Haye, 54505, Vandœuvre-lès-Nancy, France; 2 INRA UMR 1121, Agronomie et Environnement Nancy-Colmar, ENSAIA 2 avenue de la forêt de Haye, 54505, Vandœuvre-lès-Nancy, France; 3 CNRS UPR 2357 28 rue Goethe, Strasbourg, 67000, France

## Abstract

**Background:**

Furanocoumarins are molecules with proven therapeutic properties and are produced in only a small number of medicinal plant species such as *Ruta graveolens*. *In vivo*, these molecules play a protective role against phytophageous insect attack. Furanocoumarins are members of the phenylpropanoids family, and their biosynthetic pathway is initiated from *p-*coumaroyl coA. The enzymes belonging to the CYP98A cytochrome P450 family have been widely described as being aromatic *meta*-hydroxylases of various substrates, such as *p-*coumaroyl ester derivatives, and are involved in the synthesis of coumarins such as scopoletin. In furanocoumarin-producing plants, these enzymes catalyze the step directly downstream of the junction with the furanocoumarin biosynthetic pathway and might indirectly impact their synthesis.

**Results:**

In this work, we describe the cloning and functional characterization of the first CYP98A encoding gene isolated from *R. graveolens*. Using *Nicotiana benthamiana* as a heterologous expression system, we have demonstrated that this enzyme adds a 3-OH to *p-*coumaroyl ester derivatives but is more efficient to convert *p-*coumaroyl quinate into chlorogenic acid than to metabolize *p-*coumaroyl shikimate. Plants exposed to UV-B stress showed an enhanced expression level of the corresponding gene. The *R. graveolens cyp98a22* open reading frame and the orthologous *Arabidopsis thaliana cyp98a3* open reading frame were overexpressed in stable transgenic Ruta plants. Both plant series were analyzed for their production of scopoletin and furanocoumarin. A detailed analysis indicates that both genes enhance the production of furanocoumarins but that CYP98A22, unlike CYP98A3, doesn’t affect the synthesis of scopoletin.

**Conclusions:**

The overexpression of CYP98A22 positively impacts the concentration of furanocoumarins in *R. graveolens.* This gene is therefore a valuable tool to engineer plants with improved therapeutical values that might also be more resistant to phytophageous insects.

## Background

The adaptation of plants to their environment and their survival under stressing conditions, e.g., pathogenic attacks, requires secondary metabolites, such as polyphenols. These molecules are broadly distributed in the plant kingdom with more than 8,000 phenolic structures currently known, ranging from simple molecules, such as phenolic acids, to highly polymerized substances, such as tannins [1]. The furanocoumarins constitute one of these classes of polyphenols. Despite their importance in plant life, their biosynthesis remains relatively poorly documented at the molecular level. These molecules exist mainly in 4 plant families: Rutaceae, Apiaceae, Fabaceae and Moraceae where they play diverse functions in plant adaptation to the environment as phytoalexins in defense systems [2] or in plant-insect interactions [3]. These molecules also display remarkable physicochemical properties, making them potentially toxics. They can interfere in enzymatic reactions through the inhibition of cytochrome P450 (P450) enzymatic activities [4,5]. They also interact with nucleic acids through the photocycloaddition of pyrimidic bases [6]. These characteristics make furanocoumarins attractive candidates for therapeutic use. For example, furanocoumarin derivatives have been used for decades as treatments for skin diseases, such as psoriasis and vitiligo [7]. In addition, there are other applications for furanocoumarins in various therapeutic fields, such as the symptomatic treatment of multiple sclerosis [8], photochemotherapy of T cell lymphoma [9], or chemotherapy of multidrug-resistant tumors [10]. Thus, it would be beneficial to increase the production of furanocoumarins in plants to match pharmaceutical demand. To reach this goal, it is essential to understand the biosynthetic pathway of furanocoumarins, and to determine how the production of these molecules could be enhanced.

Furanocoumarin-producing plants are not model plants for the scientific community. Therefore, little is known about their genomes and the genes that encode the enzymes involved in their biosynthetic pathways. Only four genes have been functionnaly characterized so far. Two P450s, psoralen synthase and angelicin synthase, have been described and are specifically involved in the synthesis of these molecules. These synthases catalyze the transformation of marmesin and colombianetin in psoralen and angelicin, respectively [11,12]. Another study reported the identification and the characterization of a *o*-methyl transferase in *Ammi majus* that catalyzes the transformation of bergaptol into bergapten [13]. Finally, a Fe^2+^/α-ketoglutarate-dependent dioxygenase was recently identified in *Ruta graveolens*, which is able to metabolize *p*-coumaroyl coA leading to the synthesis of umbelliferone, the immediate precursor of furanocoumarins [14].

**Figure 1:**
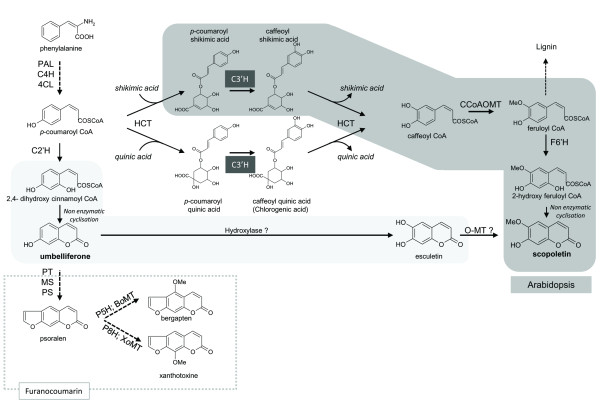
**Simplified phenylpropanoid biosynthesis pathway (PAL: phenyl ammonia lyase, C4H: cinnamate 4-hydroxylase, 4CL: 4-coumaroyl CoA ligase, HCT: hydroxycinnamoyl transferase, C3’H: cinnamoyl ester 3’-hydroxylase), C2’H: *p *-coumaroyl CoA 2’-hydroxylase, PT: prenyltransferase, MS: marmesin synthase, PS: psoralen synthase, P5H: psoralen 5-hydroxylase, B *o *MT: bergaptol *o *-methyltransferase, P8H: psoralen 8-hydroxylase, X *o *MT: xanthotoxol *o *-methyltransferase, F6’H: feruloyl CoA hydroxylase, CCoAOMT: caffeoyl CoA O-methyltransferase Black dashed arrows: multistep reaction.**

CYP98A (C3’H) are enzymes belonging to the cytochrome P450 family that catalyze the *meta*-hydroxylation of *p-*coumarate derivatives, an important step in the phenylpropanoid pathway (Figure1). This *meta*-hydroxylation is not operating on free *p*-coumaric acid, but on *p-*coumarate esterified with shikimic, quinic, tyramine or phenyllactic acids [15]. In most of the studies, these enzymes showed preferential affinity for the 5-*O*-shikimate and 5-*O*-D-quinate esters of *transp*-coumaric acid [16-18], however 4-coumaroyl-3’, 4’-dihydroxyphenyllactate and *p*-coumaroyl tyramine are also precursor molecules that form rosmarinic acid and caffeoyltyramine, respectively [18-20]. Recently, another study demonstrated a complex process of evolution of the CYP98A family to acquire new functions implicated in the mechanism of pollen development in plants [21]. Other studies have assigned roles for these enzymes in the response of the plants to various molecules, such as salicylic acid derivatives and isonicotinic acids [22], and the expression level of the corresponding genes is highly increased after UV-C treatment [23]. Therefore, the CYP98A subfamily plays a major role in the phenylpropanoid pathway, and its action is not limited to the direct synthesis of caffeoyl ester derivatives but has a larger impact on plant metabolism. 

In the present work, we describe the identification and *in vitro*/*in vivo* enzymatic characterization of a new gene encoding CYP98A22, a *p*-coumaroyl ester 3'-hydroxylase from *R. graveolens,* which constitutes the first CYP98 characterized from a furanocoumarin-producing plant. The biochemical characterization was assayed in 3 different systems: i) heterologous expression in yeast using the galactose-inducible strain pYeDP60/WAT11, ii) heterologous transient expression in the leaves of *N. benthamiana* together with the TBSV P19-silencing suppressor, and iii) stable expression in *R. graveolens* plants. Our results clearly show that CYP98A22 preferentially hydroxylates *p-*coumaroyl quinate to a greater extent than *p-*coumaroyl shikimate and, therefore seems to be involved in chlorogenic acid metabolism. Leaf exposure to UV-B light and further analyses of the expression level of *CYP98A22* revealed an increased *cyp98a22* mRNA accumulation. Finally, the analyses of the coumarin and furanocoumarin extracted from transgenic *R. graveolens* overexpressing *cyp98a22* or *cyp98a3* clearly showed an increase in the concentration of furanocoumarins in both cases whereas the accumulation scopoletin could only be observed for the CYP98A3 plants. Therefore, the work described here demonstrates that CYP98A22 can be used as a tool to modulate the furanocoumarins content in *R. graveolens.*

## Results

### Identification of a cyp98a orthologous gene in *R. graveolens*

To identify *cyp98a* genes present in furanocoumarin-producing plants, we used a PCR-based approach and the CODEHOP strategy described by Morant et al. [24]. First, we focused on the identification of genes belonging to the CYP98A subfamily. To achieve this, we performed an alignment of 9 sequences of CYP98A available in databases which allowed us to identify two peptidic consensus domains (EWAMAEL and PFGAGRR) and define degenerated primers. The PCR reactions were performed on genomic DNA extracted from young *R. graveolens* seedlings. A DNA fragment of 389 nucleotides corresponding to the internal sequence of a gene encoding a cytochrome P450 was amplified and subsequently cloned. A Genbank homology search using the Blast tool showed 89% identity at the amino acid level with a C3'H isolated from *Ocinum basilicum* (AAL99200.1). The corresponding full length open reading frame was isolated by using PCR conducted on a SMART cDNA library produced from RNA extracted from the leaves of young *R. graveolens* seedlings [14] as described in material and methods. The resulting sequence (GenBank JF799117, Additional file 1) was 1527 bp long and encoded for a 508 amino acid protein, which displayed 81% identity with the Arabidopsis CYP98A3.

### *In vitro* biochemical characterization of CYP98A22

To characterize the activity of CYP98A22, the open reading frame was cloned into the pYeDP60 plasmid and expressed in the *Saccharomyces cerevisae* WAT11 strain as described by Larbat and collaborators [11]. The P450 functional expression was determined at 450 nm using a CO differential spectrum. Unfortunately, for the yeast expressing CYP98A22, no peak could be detected with microsomes prepared from cells grown in conventional or modified culture conditions (increase of the induction length from 12 to 24 h and a decrease in the induction temperature from 28 to 18°C). Despite undetectable levels of expression, incubations were performed using the substrates previously described for the enzymes of this P450 family. We were unable to detect any significant activity using HPLC-DAD measurements with *p-*coumaroyl quinate, *p-*coumaroyl shikimate, *p*-coumaroyl tyramine and *p*-tricoumaroyl spermidine as substrates. In parallel, microsomes prepared from yeast expressing CYP98A3 were perfectly functional and consistent with the results described elsewhere, which showed that the preparation of microsomes was efficient.

**Figure 2:**
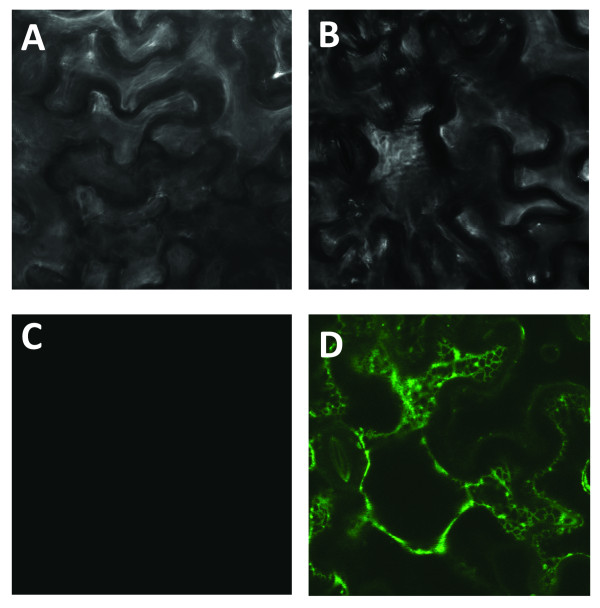
**Confocal microscopy images showing subcellular CYP98A22-YFP localization in *Nicotiana benthamiana *leaves coinfiltrated with *Agrobacterium tumefaciens *containing pBIN61-P19 and A and C) the empty pBIN-GW plasmid or B and D) pBIN-CYP98A22-YFP.** The images were recorded at 3 days post-infiltration. **A** and **B**: visible light, **C** and **D**: 488nm excitation.

The absence of CYP98A22 activity in the yeast expression system led us to produce the protein in another heterologous expression system, *i.e. Nicotiana benthamiana*, using a modified protocol of Voinnet *et al.*[25]. In an initial attempt to monitor the expression of the corresponding proteins, the YFP protein was fused to the C terminus of the gene encoding CYP98A22. The recombinant coding sequence was cloned into the binary plasmid pBIN-GW [14] and introduced into a LBA4404 *Agrobacterium tumefaciens* strain [14]. Young leaves of *N. benthamiana* were infiltrated with *A. tumefaciens* LBA4404 which was transformed with either pBIN-CYP98A22-YFP or the empty pBIN plasmid. The coinfiltration was performed along with Agrobacterium transformed with pBIN61-P19. A strong fluorescence signal was observed at the endoplasmic reticulum level in the case of the coexpression of P19 and CYP98A22-YFP (Figure2D), whereas no signal could be detected with the control empty plasmid (Figure2C). These experiments demonstrated that *N. benthamiana* was an effective system for the expression of CYP98A22. 

To assess the function of CYP98A22, a second set of experiments was conducted using a recombinant pBIN plasmid containing *cyp98a22* as a single sequence (without the presence of YFP). The agrobacteria transformed with this plasmid were coinfiltrated into *N. benthamiana* leaves along with recombinant *A. tumefaciens* containing pBIN61-P19. In parallel, as two independent controls, the inoculation of separate plants was performed with *A. tumefaciens* transformed with pBIN-CYP98A3 or with the pBIN-GW void plasmid. Four days post-inoculation, microsomes were prepared from the infiltrated *N. benthamiana* leaves as described in the material and methods section. These microsomes were tested for *in vitro* enzymatic activity with 100μM of various potential substrates, i.e., *p-*coumaroyl shikimate, *p-*coumaroyl quinate, *p-tri*coumaroyl spermidine or *p-*coumaroyl tyramine. For both proteins (CYP98A22 and CYP98A3), no metabolisation could be detected in the presence of *p-tri*coumaroyl spermidine or *p-*coumaroyl tyramine but *p-*coumaroyl shikimate and *p-*coumaroyl quinate, were transformed in two products that shared similar retention time and UV spectrum than caffeoyl shikimate and caffeoyl quinate standards, respectively. The identity of the caffeoyl products was confirmed using LC/MS analyses (see Additional files 2 and 3). No metabolization of any substrate was observed when the infiltration was performed with the void plasmid showing that the expression level of the endogenous CYP98A from *N. benthamiana* was not high enough to be taken into account. The V_*i*_/K_*m*_ and apparent K_*m*_ kinetic parameters were determined for CYP98A22 by titration of the product formed using mass spectrometry. Results showed a higher apparent affinity (K_*m*_ 3.4 times lower) as well as a higher catalytic efficiency (V_*i*_/K_*m*_ 9 times higher) for *p-*coumaroyl quinate than for *p-*coumaroyl shikimate (Table1, Additional file 4).

**Table 1:** **Kinetic parameters of CYP98A22 determined with *p- *coumaroyl quinate and *p *-coumaroyl shikimate as substrate**

**Substrate**	**Product**	***V***_***max***_	***K***_***m***_	***V***_***i***_***/ K***_***m***_
		**(pmol.min**^**-1**^**.g**^**-1**^**FW)**	**(μM)**	**(μM.pmol**^**-1**^**.min**^**-1**^**.g**^**-1**^**FW)**
*p-*coumaroyl quinate	caffeoyl quinate	0.49 +/- 0.16	7 +/- 5	0.07 +/- 0.02
*p*-coumaroyl shikimate	caffeoyl shikimate	0.19 +/- 0.03	24 +/- 7	0.008 +/- 0.002

Reactions were carried out at 28°C during 30 min in a KPi buffer 20Mm pH 7.0 as described in material and methods. Reactions were stopped with addition of 0.1% trifluoroacetic acid. Data obtained were fitted with the Michaelis-Menten equation.

### *In planta* biochemical characterization of CYP98A22

**Figure 3:**
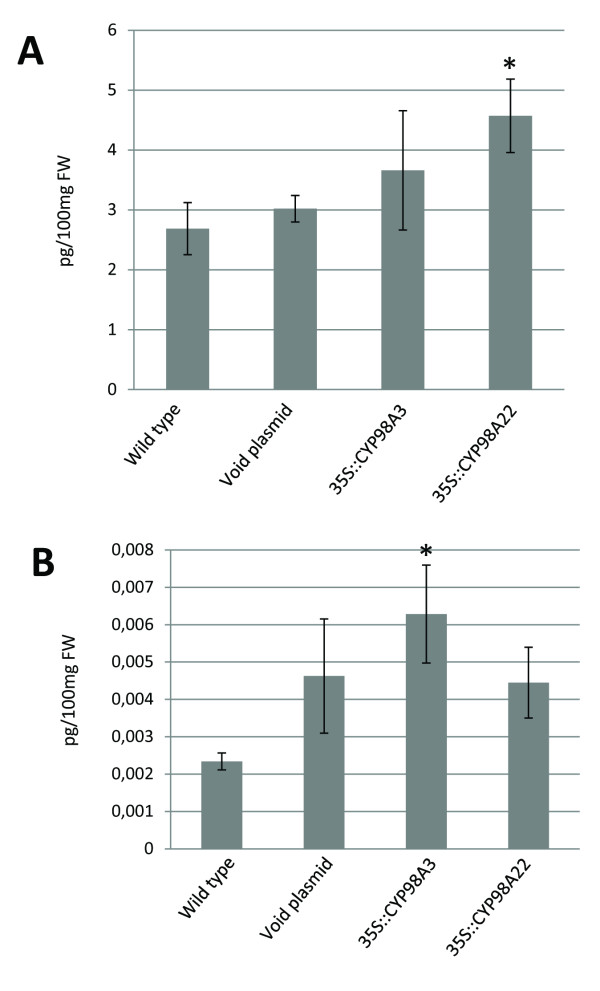
**Chlorogenic acid (A) and caffeoyl shikimic (B) content in *N. benthamiana *infiltred leaves.A**) Difference between 35S::98A22 and the wild type is statistically significant (*: Student test T statiscally significant, p < 0.05). Difference between 35S::98A3 and the wild type is statistically not significant but shows a tendency (0.1 > p > 0.05). **B**) Difference between 35S::98A3 and the wild type is statistically significant (*: Student test T statiscally significant, p < 0.05). Difference between 35S::98A22 and the wild type is statistically not significant (p > 0.05). Analyses have been done on 6 independently infiltrated leaves.

A first series of phytochemical investigations (LC/MS) were achieved on wild type leaves of *N. benthamiana* and revealed that these tissues contain a significant pool of *p-*coumaroyl shikimate and quinate. Thus, we assumed that *in planta* functional characterization of CYP98A22 could be examined following transient expression in epidermal cells, because the potential substrates of this enzyme were present. This type of approach has 2 main advantages: the accuracy of the *in vitro* results can be demonstrated and the risk of the loss of enzymatic activity during the protein purification process is reduced. To this end, we infiltrated *N. benthamiana* leaves with recombinant *A. tumefaciens* containing pBIN35S::CYP98A22, pBIN35S::CYP98A3 or an empty pBIN-GW vector. All infiltrations were analyzed along with Agrobacterium containing pBIN61-P19. The LC/MS analyses of the extracts prepared four days post-infiltration showed an increase of chlorogenic acid and caffeoyl shikimate derivatives when any *cyp98a* was transiently overexpressed (Figure3A and 3B). When comparing the accumulation of each molecule in relation with the overexpression of *cyp98a3* or *cyp98a*22, we observed that CYP98A22 is more efficient for the production of chlorogenic acid (Figure3A) whereas CYP98A3 seems to be more dedicated to the synthesis of caffeoyl shikimate (Figure3B) These results are consistent with those obtained for the *in vitro* characterization in the plant microsomes and shows that CYP98A22 is more specific for *p-*coumaroyl quinate (the precursor of chlorogenic acid).

### Over-expression of cyp98a22 in *R. graveolens*

**Figure 4:**
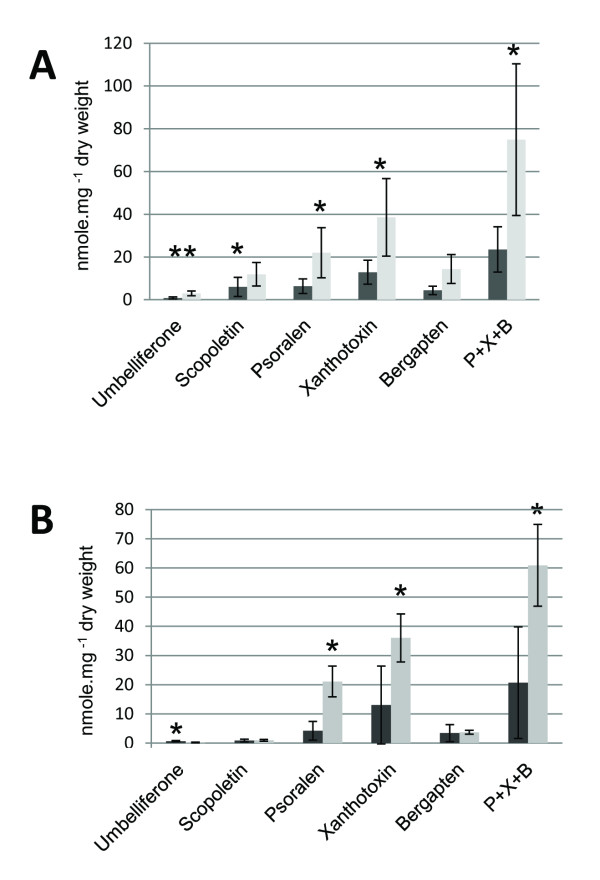
**Concentration level of umbelliferone, scopoletin, psoralen, bergaptol and xanthotoxin in *35S: cyp98a3 *, *35S: cyp98a22 *transgenic *Ruta graveolens *and wild type plants.** Black bars are corresponding to wild type plants, grey bars are corresponding to transgenic plants. The results corresponds to the mean value of analyses performed on **A**) 6 wild type plants and 9 35S::CYP98A3 transgenic plants and **B**) 4 wild type plants and 4 35S::CYP98A22 transgenic plants. *: Student test T statiscally significant (p < 0.05), **: Student test T statiscally highly significant (p < 0.01).

To establish a link between CYP98A22 and/or CYP98A3 and the synthesis of coumarins or furanocoumarins, transgenic *R. graveolens* were generated which overexpressed the corresponding gene*.* The same recombinant *A. tumefaciens* strains, used for transient expression in *N. benthamiana,* were used for stable transformation (either pBIN-CYP98A22 or CYP98A3). The transformation protocol was performed as described by Lièvre *et al.*[26]. Four 35S:CYP98A22 and nine 35S:CYP98A3 plants were generated and characterized at the molecular level using PCR directed against the *35S- cyp98a22* (the endogenous *cyp98a22* gene was not amplified) and the *cyp98a3* sequences. For each plant, the samples were divided into two halves. The first half was used to perform an RNA extraction to confirm the expression level of the transgenes using real-time PCR. The second half was used to extract phenolic molecules, which were further analyzed using HPLC. These analyses were compared with a set of extractions from wild-type plants. The content of scopoletin, umbelliferone and the three major furanocoumarins (psoralen, bergapten and xanthotoxin) present in *R. graveolens* was analyzed. For the 35S:CYP98A3 plants (Figure4A), an increase in the concentration of scopoletin and umbelliferone could be observed (2-fold and 3.6-fold respectively; p < 0.05). For the 35S::CYP98A22 (Figure4B) plants, no impact was observed for the production of scopoletin whereas an important decrease of umbelliferone (3.2 times, p < 0.05) could be highlighted. Moreover, in both cases, the results showed a statistically significant increase in the furanocoumarin content (3.2 times increase for the 35S:CYP98A3 plants (p < 0.05), and 2.9 times increase for the 35S:CYP98A22 plants (p < 0.05)) (Figure4A and B). 

### Tissue-specific expression pattern of cyp98a22 in *R. graveolens*

**Figure 5:**
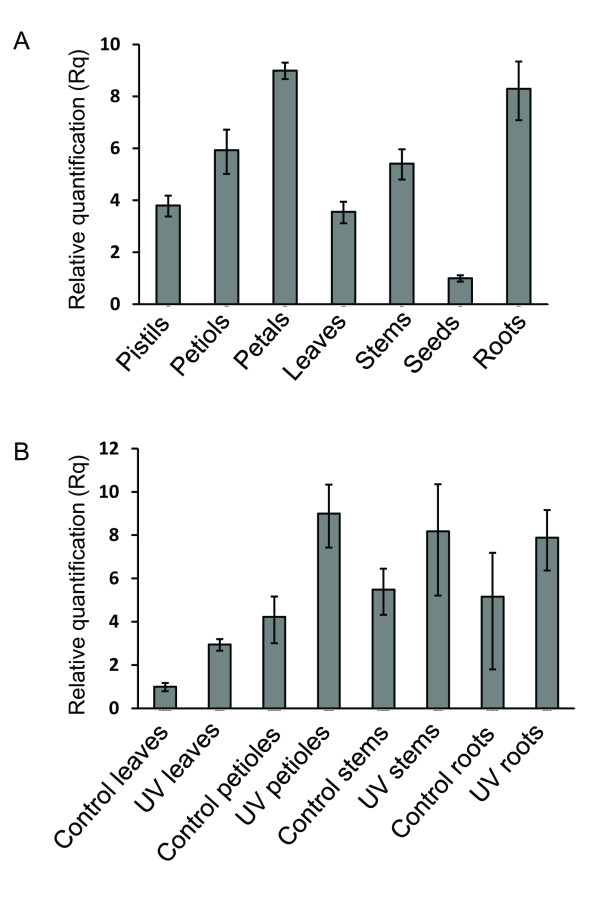
**Expression level of *cyp98a22 *in *Ruta graveolens *.** Vertical bar chart showing the results of real-time RT-PCR experiments **A**) in different organs of the plants **B**) with or without UV-B stress conditions. UV indicates UV-B induced. Control indicates UV-B non induced. (The data 2^-ΔΔCt^ were deduced using the Ct method; the error bars represent the standard errors of 3 replicates).

Total RNA was extracted from various organs of 3-month-old *R. graveolens plants* (leaves, seeds, petals, pistils, roots, and stems) and was used to establish the relative expression pattern of *cyp98a22* using a real-time PCR approach. Two sets of primers were selected for the quantification of *cyp98a22* mRNA, and the two probes gave similar results. The results showed a broad expression pattern of the mRNA (Figure5A). Indeed, mRNA was detected in all the tissues tested and was not restricted to lignified tissues. The results showed that the expression level is highest in the roots and petals and is also significantly high in the petioles and pistil. For the stems, leaves and seeds, the expression was much lower.

### UV-B treatment of *R. graveolens*

As described by different authors [14,27], the biosynthesis of furanocoumarins in *R. graveolens* plants can be enhanced through exposure to UV-B radiations*.* After a 24-hour exposure to UV-B light (312 nm), the leaves, stems, petioles and roots of *R. graveolens* plants were collected and both total RNA and furanocoumarins were extracted. As a control, the same extractions were performed on non-UV-treated plants. The LC-MS quantification was conducted for bergapten, xanthotoxin, psoralen, marmesin and umbelliferone in the treated and nontreated plants. The results showed a 3.5-fold increase of umbelliferone content in the UV-treated plants (1.4 _ 0.1 mg g^-1^ FW) compared to the control plants (0.4 _ 0.1 mg g^-1^ FW) as described in Vialart *et al.*[14]. Concerning other molecules, no statistically difference could be demonstrated. Real-time PCR experiments were performed on the same set of material as published in Vialart et al. The expression of *cyp98a22* was significantly stimulated in the UV-treated leaves (3-fold increase), whereas no statistically significant change was observed in the petioles, stems or roots (Figure5B).

## Discussion

The function of the P450 CYP98A gene subfamily has been extensively discussed in the literature for over 10 years. More than 60 different genes have been sequenced and referenced in the international P450 database (http://drnelson.uthsc.edu/cytochromep450.html) as belonging to this subfamily. Various functions have been attributed to these proteins. Their activity is classically described as a C3’H catalyzing hydroxylation in the 3' position of *p-*coumaroyl shikimate and *p-c*oumaroyl quinate [15,23,28]. However, other substrates have also been described, such as 4-*p*-coumaroyl-3' 4'-dihydroxyphenyllactate [19], *p-*coumaroyl tyramine [18] or spermidine esters [21]. Among the enzymes that metabolize *p-*coumaroyl shikimate, some were demonstrated to hydroxylate *p-*coumaroyl quinate at a lower rate and efficiency [15], while others were simply unable to metabolize both substrates [28]. In some cases, these enzymes have been described as having a larger effect on metabolism. For instance, Schoch and collaborators [17] indicated that the presence of C3’H is necessary for the biosynthesis of many divergent compounds such as, lignins, UV-absorbing pigments, antioxidants, flavors, fragrances and coumarins. Together, all these data show that this enzyme plays a central (Figure1) and pivotal role in the phenylpropanoid pathway and that it has an indirect impact on the synthesis of several molecules. In this work, we hypothesized that an orthologous gene isolated from a furanocoumarin-producing plant might have impact the synthesis of some of these molecules and play an important role in the answer to various stresses.

Little is known concerning the genome of *R. graveolens*, a furanocoumarin-producing plant. Only a few accessions (less than 80) are available in the nucleic acid or protein databases, and no information concerning a putative gene corresponding to a *cyp98a* orthologous gene was found. However, each P450 subfamily shares several conserved consensus domains which makes it possible to use sequence-based PCR approaches for cloning and identifying plant cytochrome P450 genes. Accordingly, we used a PCR approach with degenerated primers [24] to isolate a gene from the *R. graveolens* genome that potentially encodes a C3’H. The translational product of the resulting open reading frame (ORF), *cyp98a22* (Genbank JF799117), was compared with 25 peptidic sequences available in the databases. The results showed that the protein is clustering with enzymes described as classical C3’H involved in the synthesis of lignin precursor, among which the first C3’H CYP98A3 ever functionally described and not with divergent CYP98 as the *Arabidopsis thaliana* CYP98A8 and CYP98A9 which are involved in the synthesis of *N*^*10*^-monosinapoyl-*N*^*1*^*,N*^*5*^-dihydroxyferuloyl spermidine, an important constituent of pollen [21]. This first set of data seemed to indicate that this enzyme might be a conventional C3’H that potentially metabolized *p-*coumaroyl quinate and *p-*coumaroyl shikimate.

The biochemical characterization of plant cytochrome P450s using a yeast expression system [29] is often difficult due to the low abundance and instability of these membrane-bound proteins. Thus, several different heterologous systems for the expression of the P450s, including yeast, *E. coli* and baculovirus, have been described in the literature. To perform the enzymatic characterization of CYP98A22, we selected the yeast expression system described by Pompon and collaborators [29] which is extensively used for the heterologous expression and characterization of enzymatic activities of proteins belonging to the P450 family. This system is efficient for the production of certain enzymes [30] but was limited for the production of other plant enzymes. Various improvements have been proposed in the literature, including changes in the media and culture conditions [31], the modification or exchange of nucleotide sequences (partial or complete recoding of genes) [32,33] or the replacement of the membrane anchor [32][12]. A carbon monoxide differential spectrum is generally performed to assess the expression level of P450 in such a system. To achieve the expression of *cyp98a22*, several of these strategies were employed. Although no peak at 450 nm could be observed with a CO spectrum for any strategy tested, we used the prepared microsomes to conduct metabolisation tests using the following substrates: *p-*coumaroyl quinate, *p-*coumaroyl shikimate, *p-*coumaroyl tyramine, and *p-*tricoumaroyl spermidine. No transformation of any substrate could be detected, whereas the metabolisation of *p-*coumaroyl quinate and shikimate was observed in the presence of CYP98A3 yeast microsomes. This lack of expression could not been explained so far but led us to move to another protein heterologous expression system.

To determine the activity of CYP98A22, we used *N. benthamiana* as an alternative heterologous expression system. The transient transformation of the plant petals with cytochrome P450s using particle bombardment as a technique for transferring a T-DNA has been described in the literature [34,35]. The use of this system has also been reported for the functional characterization of a P450 belonging to the CYP71A subfamily involved in the biosynthetic pathway of alkaloid phytoalexins [36].

To test the efficiency of this plant heterologous expression system to allow the expression of CYP98A22, we first constructed a fusion protein comprising CYP98A22 and the yellow fluorescent protein (YFP). This recombinant ORF was placed under the control of the CaMV 35S promoter and agroinfiltrated in the epidermal cells of *N. benthamiana*. Because no fluorescence could be observed, a second set of infiltrations was performed in the presence of a plasmid allowing the expression of the Tomato Bushy Stunt Virus silencing suppressor protein P19 as described by Voinnet *et al.*[25]. In this case, the analyses of the infiltrated leaves revealed a strong fluorescence signal localized in the endoplasmic reticulum. This result is consistent with the work of Ro and collaborators who demonstrated that the cinnamate 4-hydroxylase (C4H), a cytochrome P450 enzyme of the phenylpropanoid pathway, is bound in the ER membrane [37]. Our results show that this plant system is efficient for the functional expression of CYP98A22 in the presence of the gene silencing suppressor protein.

To realize the functional characterization of the enzyme, a new set of plasmids were constructed containing the ORF of *cyp98aA22* and *cyp98a3* under the control of the 35S promoter in the pBIN vector prior to agroinfiltration to *N. benthamiana* in the presence of TBSV P19. As a first attempt, we prepared microsomes from the leaves at 4 days post-infiltration, and the enzymatic tests showed that both *p-*coumaroyl quinate and *p-*coumaroyl shikimate were hydroxylated to their caffeic counterparts by CYP98A22 and CYP98A3. However, CYP98A22 showed a much higher efficiency with *p-*coumaroyl quinate as a substrate than with *p-*coumaroyl shikimate, whereas CYP98A3 seems to preferentially metabolize *p-*coumaroyl shikimate. This first experiment revealed that the *N. benthamiana* plant system is a more efficient tool for the expression and the *in vitro* characterization of CYP98A22 than the yeast system. The use of this plant heterologous system for the expression of CYP98A22 also provides a rapid method to test the *in vivo* activity of the concerned protein. In addition to a pool of *p-*coumaroyl esters of quinate and shikimate, the dominant phenylpropanoids of tobacco is chlorogenic acid [38]. The presence of these molecules in plants provides an interesting tool to determine the activity of the overexpressed CYP98A enzymes and to highlight the natural function of these enzymes. The analysis of the metabolic profile and the quantification of chlorogenic and caffeoyl shikimic acid were performed on leaves infiltrated with recombinant agrobacteria containing pBIN-CYP98A22 and on non-infiltrated leaves. The results showed a statistically significant increase of the chlorogenic acid content.

To complete this study, we examined the tissue-specific expression pattern using real-time PCR. Indeed, as discussed above, most of the members of the CYP98A P450 subfamily catalyze efficient 3’-hydroxylation of *p-*coumaroyl shikimate and much slower synthesis of caffeoyl quinate [15,39]. Because the hydroxylation of *p*-coumaroyl-shikimate is a key step in the formation of the monolignol lignin precursor, this observed activity, along with the relatively high level of expression of most of the described C3’H in stem and vascular bundles, indicates that these genes play an important role in lignification [15,16,39]. The results we obtained in our investigation concerning the expression pattern of *cyp98a22* were somewhat different. Unlike other C3’H, *cyp98a22* displays a broad expression pattern. Strong expression was observed in the petals and roots, whereas a relatively weak expression was observed in the petioles, stems and pistils.

Together, these results show that even if CYP98A22 is phylogenetically related to CYP98A3, it might play a more complex or, at least, a different role *in planta.* Indeed, caffeoyl shikimate plays an important role in lignification, while the caffeoyl quinate derivatives, such as chlorogenic acid, are described as growth regulators, disease resistance factors, antioxidants and compounds affecting the organoleptic quality of fruits [40,41]. The ability of CYP98A22 to be more dedicated to the synthesis of chlorogenic acid constitutes an evidence of its involvement in the stress response.

To explore this hypothesis, plantlets were exposed to UV-B light for 24 hours as described by Vialart *et al.*[14]. Altough the analysis of the furanocoumarin content showed no increase in comparison with the non-treated plants, the UV-B elicitation stimulated the synthesis of umbelliferone, which is a precursor of this pathway. Moreover, these analyses demonstrated that the expression level of *cyp98a22* gene was strongly increased (3-fold) in UV-treated leaves as compared with non-treated leaves. Recently, the elicitation experiments were utilized in *Cynara cardunculus* to induce the expression of *cyp98a49* using UV-C light [23]. Although this does not provide irrefutable evidence, these elements are consistent with the hypothesis that CYP98A22 is involved in responses against several stresses in *R. graveolens*. These preliminary results must now be explored in depth.

Scopoletin is an important defense compound ubiquitously found in higher plants for which two biosynthetic routes have been described (Figure1). A first route, operating through feruloyl-CoA was demonstrated by Kai and collaborators [42] in Arabidopsis, which implies the conversion of *p*-coumaroyl-shikimic acid into caffeoyl-shikimic acid by CYP98A3, upstream to feruloyl-CoA [43]. Scopoletin content was dramatically decreased in knock-out *cyp98a3* mutants, confirming the role of the Arabidopsis C3’H in the synthesis of this compound. An alternate route to scopoletin was proposed in plants such as *Daphne mezereum* L [43] and *Agathosma puberula*[44] where umbelliferone and esculetin are the direct precursors of scopoletin. It is unestablished in *R. graveolens* if the two pathways are coexisting or if only one of the two is prevalently operating. To assess the involvement of CYP98A enzymes in the synthesis of scopoletin in *R. graveolens*, transgenic Ruta plants overexpressing either *cyp98a3* or *cyp98a22* where generated. The analysis done on the *in vitro* plants highlighted a statistically significant increase of the scopoletin content in 35S::CYP98A3 plants whereas plants overexpressing *cyp98a22* did not show any modification in scopoletin content when compared to control plants. The results obtained on CYP98A3 are consistent with the findings of Kay *et al.*[45] who described the key-role of C3’H in the synthesis of scopoletin in Arabidopsis. In parrallel, the results obtained on CYP98A22 fit well with our *in vitro* investigations obtained with *N. benthamiana*, which demonstrate the preferential role of this enzyme in the synthesis of chlorogenic acid *vs* caffeoyl-shikimate, the precursor of scopoletin in the first biosynthetic route described above. The analyses were extended to other coumarin which led us to show that the overexpression of both CYP98A impact significantly the concentration of furanocoumarins in transgenic plants. This constitute an unexpected result because C3’H is not directly involved into the synthesis of furanocoumarins (Figure1). The exact reason for this increase in furanocoumarin content remains unknowned and will require further metabolomic investigations to be elucidated.

## Conclusions

The results described in this work showed that members of the CYP98A family, even if sharing high sequence homologies, don’t have the same function in all the plants. Indeed, CYP98A22 seems to be more dedicated to the synthesis of chlorogenic acid in *R. graveolens*. As mentioned previously, furanocoumarins are molecules of pharmaceutical interest for humans. *In planta*, these molecules have been described in plant defense against phytophageous insect attacks. The selection of plants synthesizing higher amounts of these molecules would be useful to facilitate their production. According to our results, CYP98A22 is a good candidate for this purpose: it has an impact on the synthesis of furanocoumarins in *R. graveolens*. These properties can be used in a metabolic engineering strategy to enhance the production of molecules of therapeutic interest. This work also confirms that the study of metabolic networks opens a wide field of investigation for the production of plants of interest for pharmaceutical applications.

## Methods

### cDNA library

The total RNA was extracted from the *Ruta graveolens* plantlets using the Plant Rneasy kit (Qiagen). The cDNA library was prepared as described by Vialart et al. [14].

### Amplification and cloning of CYP98A from *R. graveolens*

#### Design of CODEHOP primers to amplify CYP98A

Clustal W was used to perform a peptidic alignment between *cyp98a*1 (AF029856), *cyp98a*2 (AF022458), *cyp98a*3 (AC002409), *cyp98a*6 (AB017418), *cyp98a*8 (AC011765), *cyp98a*9 (AC011765), *cyp98a*13 (AAL99200), *cyp98a*19 (AY064170), and *cyp98a*20 (AY065995). The specific primers were directed against the EWAMAEL CYP98A sequence and the large PFGAGRR P450 consensus domain to generate 98-11DIR: 5’-GAGTGGGCTATGGCTGARHTNRT-3’ and 98-1R: 5’-CCTCCTGCCNGCNCCRAANGG-3’. The 5’ ends of the primers were not degenerate, and the codon usage was based on *Citrus sinensis,* which also belongs to the *Rutaceae* family. The 3’ end was completely degenerate. The PCR conditions were set as described in a previous study [24], and the starting hybridization temperature was 70°C and decreased by 1°C at each step.

#### 5’ and 3’ end amplification

The CDS III and SMART IV primers (Smart cDNA Library Construction Kit, Clontech) designed against λTriplEx were used in association with primers designed to the internal sequence of the partial *cyp98a22* gene amplified using the CODEHOP approach. *cyp98a22* was amplified using PCR (98FLREV: 5’-GGGGTACCTTACAAATCAGCAGCAACACGTTT-3’, 98FLDIR: 5’-GGGGTACCATGGGTCTCCCACTCATCCC-3’), and a *Kpn*I restriction site was inserted at the 5’ and 3’ ends. The gene encoding *cyp98a*3 was amplified from the pYeDP69-CYP98A3 plasmid [15], using the primers 98A3DIRBam: 5’-GGGATCCATGTCGTGGTTTCTAATAGCG-3’ and 98A3REVEco: 5’-GGAATTCTTACATATCGTAAGGCACGCG. The PCR was conducted using the following conditions: 94°C for 5 min, 30 cycles of 94°C for 15 sec, 50°C for 15 sec, and 72°C for 90 sec followed by a final extension step at 72°C for 10 min. The PCR fragments were introduced into the pCR8-GW-TOPO-TA vector according to the manufacturer’s recommendations.

### Construction of pYeDP60 recombinant plasmids

The recombinant pCR8 plasmids containing the *cyp98a22* sequence were inserted into the pYeDP60 yeast expression plasmid [29]. The empty pYeDP60 and pCR8 plasmids containing *cyp98a22* were digested with *Kpn* I and were purified following agarose gel electrophoresis using the MinElute Gel Extraction Kit (Qiagen). The *cyp98a22* inserts and the pYeDP60 vector were mixed and ligated using the T4 DNA ligase from Invitrogen. The resulting clones obtained in *E. coli* cells were examined using colony PCR to determine the insertion and orientation of the coding sequence.

### Construction of the YFP fusion proteins and cloning of the cyp98a22 coding sequences in plant vectors

The design of the *cyp98a22* C-terminal fusion in frame with a fluorescent reporter was a two-step procedure. The first set of the PCR reactions was designed to yield *cyp98a22* with a 18-nucleotide 3’ end overlapping the first 18 nucleotides of *YFP* (forward primer 98FLDIR, reverse primer 98A22YFPREV: 5’-CCTTGCTCACCATGTGGCGACCGGTACCCCCCAAATCAGCAGCAAC-3’) and the *YFP* sequence with an additional sequence at the 5’ end corresponding to the last 18 nucleotides from *cyp98a22* (forward primer 98A22YFPDIR: 5’- GTTGCTGCTGATTTGGGGGGTACCGGTCGCCACATGGTGAGCAAGG-3’ and reverse primer YFPREV: 5’-TTACTTGTACAGCTCGTCCATGCC-3’). In a second set of PCR reactions, the products from the previous PCR were mixed, and the amplification was performed using the CYP98A22 direct primer and the YFP reverse primer to generate the chimeric *cyp98a22-YFP* fusion gene. The recombinant sequence was inserted into the pCR8 vector (Invitrogen) according to the manufacturer’s recommendations.

### Subcloning in the pBIN-GW plasmid

The genes inserted in the pCR8 plasmid were transferred to the pBIN-GW plasmid using LR recombination as recommended by Invitrogen. The pBIN recombinant plasmids were introduced into the LBA4404 *A. tumefaciens* strain ([46]), and the resulting clones were used for the transient expression of *N. benthamiana* leaves or the stable transformation of *R. graveolens.*

### Yeast expression

The *Saccharomyces cerevisiae* WAT11 strain were transformed as described by Pompon [29] using the different pYeDP60 recombinant plasmids. The expression of the corresponding proteins and preparation of microsomes were performed as described by Larbat et al. [11].

### Transient expression in *N. benthamiana*

For *N. benthamiana* transient protein expression, 4- to 6-week-old plants were transiently transfected with *A. tumefaciens* containing pBIN 35S:98A3, pBIN 35S:98A22, pBIN 35S:98A22-YFP and pBIN61-P19 in accordance with a previously published protocol [47]. A volume of 5 mL of overnight *A. tumefaciens* culture was pelleted, washed three times with water and resuspended in water. The leaves were coinfiltrated with Agrobacterium (at a final OD_600_ of 0.2) containing one of the pBIN-98 constructs and *Agrobacterium* (at a final OD_600_ of 0.4) containing pBIN61-P19. The infiltration was performed in the lower epidermis using a 1-ml syringe (without a needle) and gentle pressure.

### Fluorescence analysis in infiltrated *N. benthamiana* leaves

Fluorescence (in the form of a fusion protein) was observed 96 hours postinoculation. For the observation of the fluorescent proteins using confocal microscopy, the leaf disks were excised, mounted between slides and coverslips, and vacuum infiltrated using water. The leaf discs were observed using a laser scanning confocal microscope (Fluoview, FV10i, Olympus). The excitation and emission wavelengths were 488 nm and 505 to 545 nm for YFP, respectively. The images presented are single focal sections. The image processing was performed using ImageJ (National Institutes of Health, http://rsb.info.nih.gov/ij/) and Photoshop 6.0 (final image assembly; Adobe Systems, San Jose, CA).

### Plant microsomes preparation and enzymatic activity test

Approximately 4 g of fresh leaves were harvested at 96 hours postinoculation, frozen in liquid nitrogen and ground in a mortar. The cell debris was resuspended in 0.1 M of KPi buffer (pH 7.0) containing a cocktail of protease inhibitors (Roche) and centrifuged for 30 min at 10,000 g. The supernatant was filtered through miracloth and centrifuged for 1 h at 100,000 g using a SORVALL WX80 ultracentrifuge. The resulting supernatant was carefully removed, and the pellet was resuspended in 200 μl of 0.1 M KPi buffer (pH 7.0). All the microsomal preparations were prepared at 4°C.

The enzymatic syntheses of *p-*coumaroyl quinate and *p-*coumaroyl shikimate were performed as described by Morant *et al.*[18]. The enzymatic assays were conducted as described by Larbat and collaborators [11]. Microsomes overexpressing CYP98A22 were incubated with 0.2 mM NADPH in 20 mM sodium phosphate buffer (pH 7.0) containing 100 μM *p-*coumaroyl shikimate or *p-*coumaroyl quinate and *p-*coumaroyl tyramine or tricoumaroylspermidine. The reaction was performed for 30 min at 28°C and 0.1 M HCl were added to terminate the reaction. The range of substrate used for the determination of kinetic parameters was 4μM, 5, 7.5, 10, 20, 30, 50μM for *p-*coumaroyl shikimate and 1μM, 2, 4, 5, 7.5, 10, 20, 30μM for *p-*coumaroyl quinate. The reaction mix was centrifuged for 30 min at 10,000 g, and 50 μl of the supernatant was analyzed using reverse phase HPLC (LiChrospher 100 RP-18 column) coupled with mass spectrometry. The HPLC program was set as follows: buffer A (H_2_O, 0.1% formic acid) and buffer B (100% MeOH, 0.1% formic acid); 5 min with 10% isocratic in buffer B followed by a linear gradient from 10 to 80% buffer B for 15 min and final a washing step. The absorbances of the hydroxylated products were measured at 330 nm with a diode array detector. The identity of the metabolized products caffeoyl shikimate and quinate were analyzed using an electrospray mass spectrometer in the negative ion mode as described by Matsuno et al. [21]. Apparent K_*m*_ values were measured by fitting the data obtained for both substrates to the Michaelis-Menten equation using the software SigmaPLOT 12.0 (Systat Software Inc).

### Chlorogenic acids and caffeoyl shikimic derivatives extracted from *N. benthamiana* and quantification

Four days postinfiltration, 6 independently infiltrated leaves were collected and crushed separately in liquid nitrogen using a mortar. A total of 0.1 g of each preparation was collected, and the quinic derivative molecules were extracted in 1 ml of 80% MeOH, vortexed vigorously for 1 min and centrifuged at 13,000 g for 20 min. The resulting supernatant was removed and evaporated to dryness. The solid extract was then resuspended in 100% MeOH prior to HPLC analysis. The chlorogenic acids were followed and identified as described by Hoffmann et al. [48]. The quantification of the esters was performed in comparison with taxifolin, an external standard, which was added at the beginning of the extraction procedure.

### LC/MS analyses of the caffeoyl derivatives

The HPLC-MS system consisted of a binary solvent delivery pump and a linear ion trap mass spectrometer (LTQ-MS, Thermo Scientific, San Jose, CA, USA). The LTQ was equipped with an atmospheric pressure ionization interface operating in the ESI negative ion mode. The data were processed using Xcalibur software (version 2.1). The operational parameters of the mass spectrometer are given below. The spray voltage was 4.5 kV and the temperature of the heated capillary was set at 300°C. The flow rates of sheath, auxiliary and sweep gases were set (in arbitrary units min-1) to 40, 10, and 10, respectively. The capillary voltage was set at -36 V, the split lens was set at 44 V and the front lens was set at 3.25 V. All the parameters were optimized by infusing a standard solution of isopimpinelline (0.1 g L^-1^) in mobile phase [water + acetic acid 0.1% / methanol + acetic acid 0.1% (90/10)] at a flow rate of 5 μl min^-1^. The caffeoylshikimic acid isomers were monitored through MS^2^ (335)-specific and full (100-400 m/z) scans. Standard solution of caffeoyl quinate and caffeoyl shikimate were used for quantification.

### Plant Material and UV-B treatments

The *R. graveolens* seeds were germinated on soil. The experimental setup under glasshouse conditions was similar to that described by Vialart and collaborators [14].

### Real-Time PCR

To examine tissue-specific gene expression, the total mRNA was extracted from 100 mg of fresh tissues from the leaves, roots, stems, petioles, petals, seeds, and flowers of *R. graveolens* plants using the RNeasy plant-mini-kit (Qiagen). A high-capacity RNA-to-cDNA master mix (Applied Biosystems) was used for the first-strand cDNA synthesis. A total of 1 μg of RNA from each organ was reverse-transcribed using oligo(dT)_17_ as a primer. The obtained cDNA samples were diluted 1:100 before use. Real-time PCR was performed using the following procedure. Specific 16S ribosomal RNA primers from *R. graveolens* were used as an internal control for data normalization (Rg16SDIR: 5’-CATTCGGCCCGTCTTGAA-3’ forward and Rg16SRev: 5’-CCGTTGACTCGCACACATGT-3’). Two pairs of primers were designed for the quantification of *cyp98a22*. The following sequences were used for the first set of primers: 98RTPCRDIR1: 5’-CACGGAGTTGGCGAAGGA-3’, 98RTPCRREV1 5’-GGTGCCTGTCAGCCAATTG-3’. The following sequences were used for the second set of primers: 98RTPCRDIR2: 5’-ACAGCAGAGTGGGCAATGG-3’, 98RTPCRREV2 5’-CCTGTGCTTTGTGTTGCACTCTA-3’. The three sets of probes were designed using the Primer Express software version 3.0 (Applied Biosystems), and their efficiency was determined using the standard linear curve method. The relative transcript level of the same gene in various organs was expressed as the log2 ratio of the 16S normalized transcript levels in a given organ in comparison with the average expression of the gene in all the organs tested.

### Plant stable transformation

The transformation of *R. graveolens* was performed as described previously [26] The regenerated transgenic plants were characterized at the molecular level using a PCR approach. The genomic DNA was extracted using the Plant DNeasy Kit (Qiagen). The PCR amplification was performed using specific primers. The forward primer was designed against the 35S promoter. This primer was the same for all the tested plants (35SDIR100: 5'-CACTATCCTTCGCAAGACCCTTCCTCTATATAAGGAA-3'). To determine the presence of transgenic *cyp98a22* and *cyp98a3*, we used the same reverse primers that were used to perform the cloning steps described above.

### Phylogenetic analysis

25 sequences of previously described CYP98A gene were aligned using the Clustal X software. An additional sequence of a divergent CYP was added to the alignment as an outgroup sequence. A molecular phylogenetic tree was generated from this alignment using the neighbor-joining method. The tree was visualized using the TreeView software.

## Competing interest

There are no competing interests either financial or not financial concerning the work described in the present manuscript.

## Authors’ contributions

KF performed the yeast and plant transient expression and the enzymatic characterization. OA conducted the metabolomic and transcriptomic analysis and the YFP fusion construction. DS constructed the transgenic *Ruta graveolens* and conducted the corresponding analysis. VG treated the *R. graveolens* with UV and performed the corresponding analysis. PU prepared and synthesized the cinnamoyl ester derivatives. DW participated to the design of the work with the yeast expression system (WAT11-CYP98A3) HA conducted the plylogenetic analysis and the isolation and cloning of the gene. HA and BF conceived the study, participated in its design. AH and BF coordinated and finalized the written manuscript. All authors read and approved the final manuscript.

## Supplementary Material

Additional file 1 **A) Alignment done on 9 CYP98A (CYP98A1 (AF029856) ; CYP98A2 (AF022458); CYP98A3 (AC002409); CYP98A6 (AB017418); CYP98A8 (AC011765); CYP98A9(AC011765); CYP98A13 (AAL99200); CYP98A19 (AY064170); CYP98A20 (AY065995).** Only the C-terminal ends of the proteins are represented. The conserved sequences used to design the CODEHOP primers are highlighted in yellow. B) Nucleotidic and peptidic sequence of CYP98A22 (Genbank JF799117). Click here for file

Additional file 2 **Identification of the metabolization product of p-coumaroyl quinate.** A) MS analysis of the metabolization product: B) MS analysis of caffeoyl quinate; C) MS^2^ analysis of the metabolization product: D) MS^2^ analysis of caffeoyl quinate. Click here for file

Additional file 3 **Identification of the metabolization product of *p-*coumaroyl shikimate.** A) MS analysis of the metabolization product: B) MS analysis of caffeoyl shikimate; C) MS^2^ analysis of the metabolization product: D) MS^2^ analysis of caffeoyl shikimate. Click here for file

Additional file 4 **Measurement of apparent K**_***m***_**of CYP98A22.** Reaction rates *versus* different concentrations of *p-*coumaroyl shikimate (4μM-50μM) (A) and of *p-*coumaroyl quinate(1μM-30 μM) (B). Apparent K_*m*_ values were measured by fitting the data to the Michaelis-Menten equation using the software SigmaPLOT 12.0. Click here for file

## Author details

^1^Université de Lorraine UMR 1121, Agronomie et Environnement Nancy-Colmar, ENSAIA 2 avenue de la forêt de Haye, 54505, Vandoeuvre-lès-Nancy, France. ^2^INRA UMR 1121, Agronomie et Environnement Nancy-Colmar, ENSAIA 2 avenue de la forêt de Haye, 54505, Vandoeuvre-lès-Nancy, France. ^3^CNRS UPR 2357 28 rue Goethe, Strasbourg 67000, France.
