# Supplementary material for: CYP98A22, a phenolic ester 3’-hydroxylase specialized in the synthesis of chlorogenic acid, as a new tool for enhancing the furanocoumarin concentration in Ruta graveolens
Source: BMC Plant Biol. 2012 Aug 29;12:152. doi: 10.1186/1471-2229-12-152 (PMC3493272; doi:10.1186/1471-2229-12-152)
Supplement: Additional file 1 — A) Alignment done on 9 CYP98A (CYP98A1 (AF029856) ; CYP98A2 (AF022458); CYP98A3 (AC002409); CYP98A6 (AB017418); CYP98A8 (AC011765); CYP98A9(AC011765); CYP98A13 (AAL99200); CYP98A19 (AY064170); CYP98A20 (AY065995). Only the C-terminal ends of the proteins are represented. The conserved sequences used to design the CODEHOP primers are highlighted in yellow. B) Nucleotidic and peptidic sequence of CYP98A22 (Genbank JF799117). [file 1471-2229-12-152-S1.doc]

**A)**

....|....| ....|....| ....|....| ....|....| ....|....|

355 365 375 385 395

CYP98A1 -----AKQHF VDALFTLKQQ YDLSEDTVIG LLW------- ---DMITAGM

CYP98A2 -----AKQHF VDALLTLQDK YDLSEDTIIG LLW------- ---DMITAGM

CYP98A3 -----AKQHF VDALLTLKDQ YDLSEDTIIG LLW------- ---DMITAGM

CYP98A6 -----TKQHF VDALLTLQKQ YDISEDTIIG LLW------- ---DMIAAGM

CYP98A8 --------GF VRKLLVLKEQ KELSEETVGG LVW------- ---NMLTAGA

CYP98A9 --------CF VQSLLELKEK DELTEETVMG LVW------- ---NMLTAGA

CYP98A13 -----AKQHF FDALLTLKDK YDLSEDTIIG LLW------- ---DMITAGM

CYP98A19 -----AKQHF VDALLTLQEK YDLSEDTIIG LLW------- ---DMITAGM

CYP98A20 -----TKNHF VDALLTLQKE YDLSDDTVIG LLW------- ---DMISAGM

....|....| ....|....| ....|....| ....|....| ....|....|

405 415 425 435 445

CYP98A1 DTTVISVEWA MAELVRNPRV QKKLQEELDR VVGRD---RV MLETDFQNLP

CYP98A2 DTTAISVEWA MAELIRNPRV QQKVQEELDR VIGLE---RV MTEADFSNLP

CYP98A3 DTTAITAEWA MAEMIKNPRV QQKVQEEFDR VVGLD---RI LTEADFSRLP

CYP98A6 DTATISTEWA MAELVRNPRV QRKAQEELDR VVGPD---RI MTEADVP---

CYP98A8 DTTAVVIEWA MAEMIKCPTV QEKAQQELDS VVGSE---RL MTESDIPILP

CYP98A9 DTTAITIEWA MAEMIRCPTV KEKVQDELDS VVGSG---RL MSDADIPKLP

CYP98A13 DTTAISVEWA MAELIKNPRV QQKAQEELDR VIGYE---RV MTELDFSNLP

CYP98A19 DTTAITVEWA MAELVRNPRI QQKAQEEIDR VVGRD---RV MNETDFPHLP

CYP98A20 VTTTITVEWA MAELVRNPRV QQKVQEELDR VVGSD---RV MTEADIPNLP

....|....| ....|....| ....|....| ....|....| ....|....|

455 465 475 485 495

CYP98A1 YLQAVVKESL RLHPPTPLML PHKASTNVKI GG--YDIPKG ANVMVNVWAV

CYP98A2 YLQCVTKEAM RLHPPTPLML PHRANANVKV GG--YDIPKG SNVHVNVWAV

CYP98A3 YLQCVVKESF RLHPPTPLML PHRSNADVKI GG--YDIPKG SNVHVNVWAV

CYP98A6 --KSI----- ---------- ---------- ---------- ----------

CYP98A8 YLQCVVKEAL RLHPSTPLML PHKASETVWV GG--YKVPKG ATVYVNVQAI

CYP98A9 FLQCVLKEAL RLHPPTPLML PHKASESVQV GG--YKVPKG ATVYVNVQAI

CYP98A13 YLQCVAKEAL RLHPPTPLML PHRSNSNVKI GG--YDIPKG SNVHVNVWAV

CYP98A19 YLQCITKEAL RLHPPTPLML PHKATQNVKI GG--YDIPKG SNVHVNVWAI

CYP98A20 YLQCVTKECF RMHPPTPLML PHKASTNVKI GG--YDIPKG ATVSVNVWAL

....|....| ....|....| ....|....| ....|....| ....|....|

505 515 525 535 545

CYP98A1 ARDPKVWSNP LEYRPERFL- ---EENIDIK GSDFRVLPFG AGRRVCPGAQ

CYP98A2 ARDPAVWKDP LEFRPERFL- ---EEDVDMK GHDFRLLPFG SGRRVCPGAQ

CYP98A3 ARDPAVWKNP FEFRPERFL- ---EEDVDMK GHDFRLLPFG AGRRVCPGAQ

CYP98A6 ---------- ---------- ---------- ---------- ----------

CYP98A8 GRDPANWINP YEFRPERFL- ---QEETDVK GRDFRVLPFG SGRRMCPAAQ

CYP98A9 ARDPANWSNP DEFRPERFL- ---VEETDVK GQDFRVLPFG SGRRVCPAAQ

CYP98A13 ARDPAVWKNP CEFRPERFL- ---EEDVDMK GHDFRLLPFG AGRRVCPGAQ

CYP98A19 ARDPAVWKDP VTFRPERFL- ---EEDVDIK GHDYRLLPFG AGRRICPGAQ

CYP98A20 ARDPAVWKNP LEFRPERFQ- ---EEDIDMK GTDYRLLPFG SGRRICPGAQ

**B)**

atgggtctcccactcatcccactatcgctcatcttcaccgtccttgcatacaacctctaccaacggctgagattcaagctcccgccaggccctcgtcctctgcctatcgtcggaaacctctaccacgtcaagccggtgaggttccggtgctacgacgaatgggctcaccactacgggccgatcatttcggtgtggttcggttccattttgaacgttgtcgtgtccaacacggagttggcgaaggaggtgctgaaggagcatgaccagcaattggctgacaggcaccggagccgatcagctgccaagttcagcagagacgggaaggacctcatttgggccgattacgggcctcattacgtcaaggttcgtaaagtgtgtacgctcgagctttttacgccgaagagactcgaggcgatgaggccgatcagagaagacgaagtgactgccatggttgaatccattttcaaagactcaactgatcctcaaaattatgggaagagcttgacagtgaaaaagtatttgggagcagtggcattcaacaacataacgaggctagcatttgggaagagatttgtgaattcagaaggtgtgatggacgaacaaggccaagaattcaaggcaattgtggccaatgggttgaagctaggagcatcgctggccatggctgancacatcccatggcttcgctggatgttcccgttggaggaagaagccttcgccaagcacggggagcaccgggaccgcctcactcgagctatcatggaagagcacacactcgctcggcagaagagcggcggtgccaagcaacattttgtggatgctttgcttacgctccaagaaaagtatgacctaagtgaagacaccatcattggcctcttatgggacatgatcacagcaggaatggacacaactgcgatcacagcagagtgggcaatggcagagctcatcaagaaccctagagtgcaacacaaagcacaggaggaactagatcgtgtggtgggtcttgaacgtgtgttgacagaaccaggtttctcaaaccttccatacttgcaagctgtggccaaggaggctctgaggttgcacccaccaactcccctgatgctccctcaccgcgccaacgccaacgtcaagataggtggctacgacattccaaaaggatcaaacgttcacgttaacgtgtgggcagtagctagggatccggctgtctggaagaaccccttggagttccggcctgagaggtatttcgaggaggatgtggacatgaaaggacatgattttaggttactgccgttcggggctggtagaagagtctgcccaggtgcacagcttggaatcaatttggtaacgtcaatgctggggcatctactgcaccattttacttgggtgccaccaccaggagtggtgcccgaggaaattgacatggctgaaaatcctggattggtaacctatatgaagacaccattacaggctgtggcaactcctaggcttccttcacaattgtacaaacgtgttgctgctgatttgtaa

MGLPLIPLSLIFTVLAYNLYQRLRFKLPPGPRPLPIVGNLYHVKPVRFRCYDEWAHHYGPIISVWFGSILNVVVSNTELAKEVLKEHDQQLADRHRSRSAAKFSRDGKDLIWADYGPHYVKVRKVCTLELFTPKRLEAMRPIREDEVTAMVESIFKDSTDPQNYGKSLTVKKYLGAVAFNNITRLAFGKRFVNSEGVMDEQGQEFKAIVANGLKLGASLAMAXHIPWLRWMFPLEEEAFAKHGEHRDRLTRAIMEEHTLARQKSGGAKQHFVDALLTLQEKYDLSEDTIIGLLWDMITAGMDTTAITAEWAMAELIKNPRVQHKAQEELDRVVGLERVLTEPGFSNLPYLQAVAKEALRLHPPTPLMLPHRANANVKIGGYDIPKGSNVHVNVWAVARDPAVWKNPLEFRPERYFEEDVDMKGHDFRLLPFGAGRRVCPGAQLGINLVTS

MLGHLLHHFTWVPPPGVVPEEIDMAENPGLVTYMKTPLQAVATPRLPSQLYKRVAADL

Supplemental data:

A) Alignment done on 9 CYP98A (CYP98A1 (AF029856) ; CYP98A2 (AF022458) ; CYP98A3 (AC002409) ; CYP98A6 (AB017418) ; CYP98A8 (AC011765) ; CYP98A9(AC011765) ; CYP98A13 (AAL99200) ; CYP98A19 (AY064170) ; CYP98A20 (AY065995). Only the C-terminal ends of the proteins are represented. The conserved sequences used to design the CODEHOP primers are highlighted in yellow.

B) Nucleotidic and peptidic sequence of CYP98A22 (Genbank JF799117)
