# Supplementary figures and images for: CYP98A22, a phenolic ester 3’-hydroxylase specialized in the synthesis of chlorogenic acid, as a new tool for enhancing the furanocoumarin concentration in Ruta graveolens
Source: BMC Plant Biol. 2012 Aug 29;12:152. doi: 10.1186/1471-2229-12-152 (PMC3493272; doi:10.1186/1471-2229-12-152)

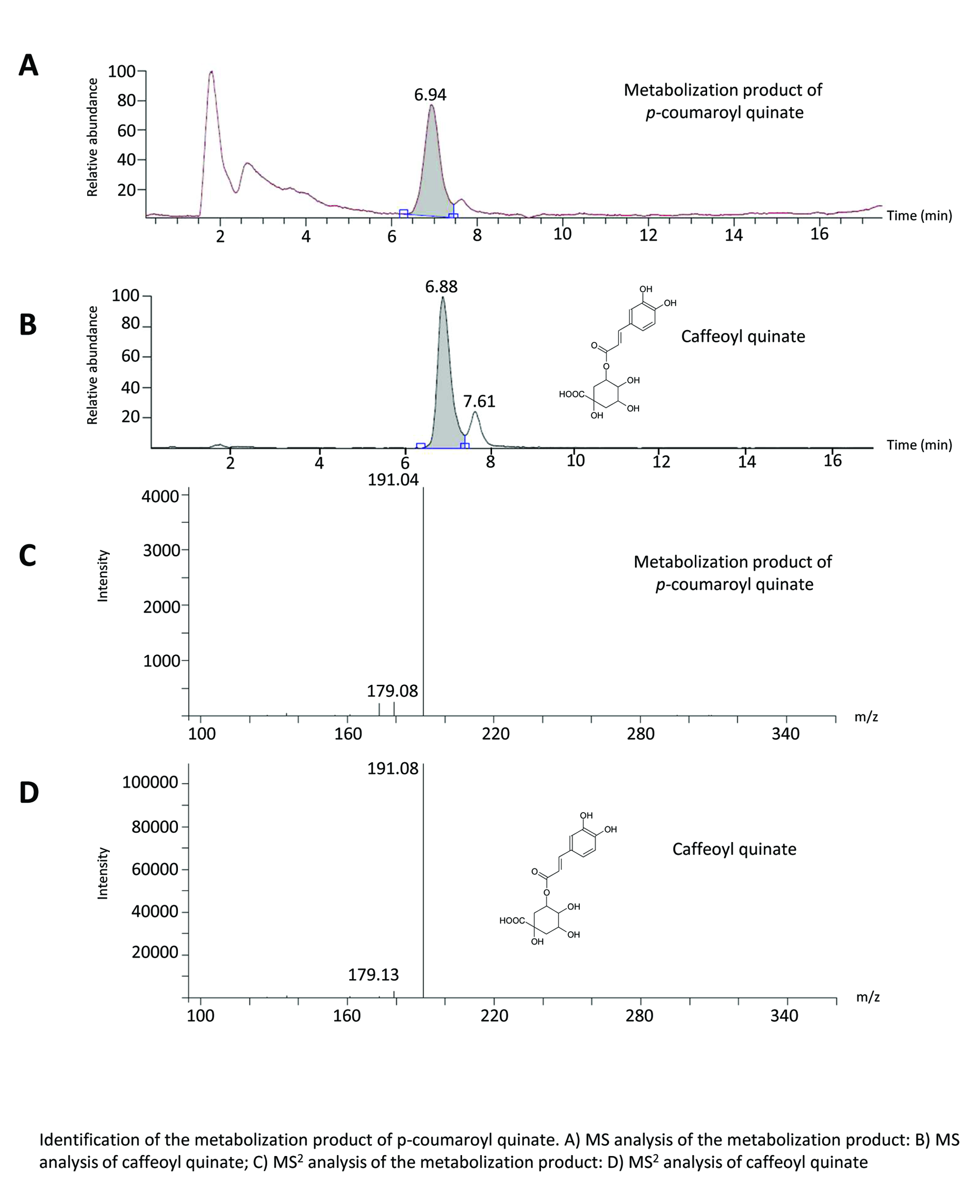

Supplement: Additional file 2 — Identification of the metabolization product of p-coumaroyl quinate. A) MS analysis of the metabolization product: B) MS analysis of caffeoyl quinate; C) MS2 analysis of the metabolization product: D) MS2 analysis of caffeoyl quinate. [file 1471-2229-12-152-S2.tiff]

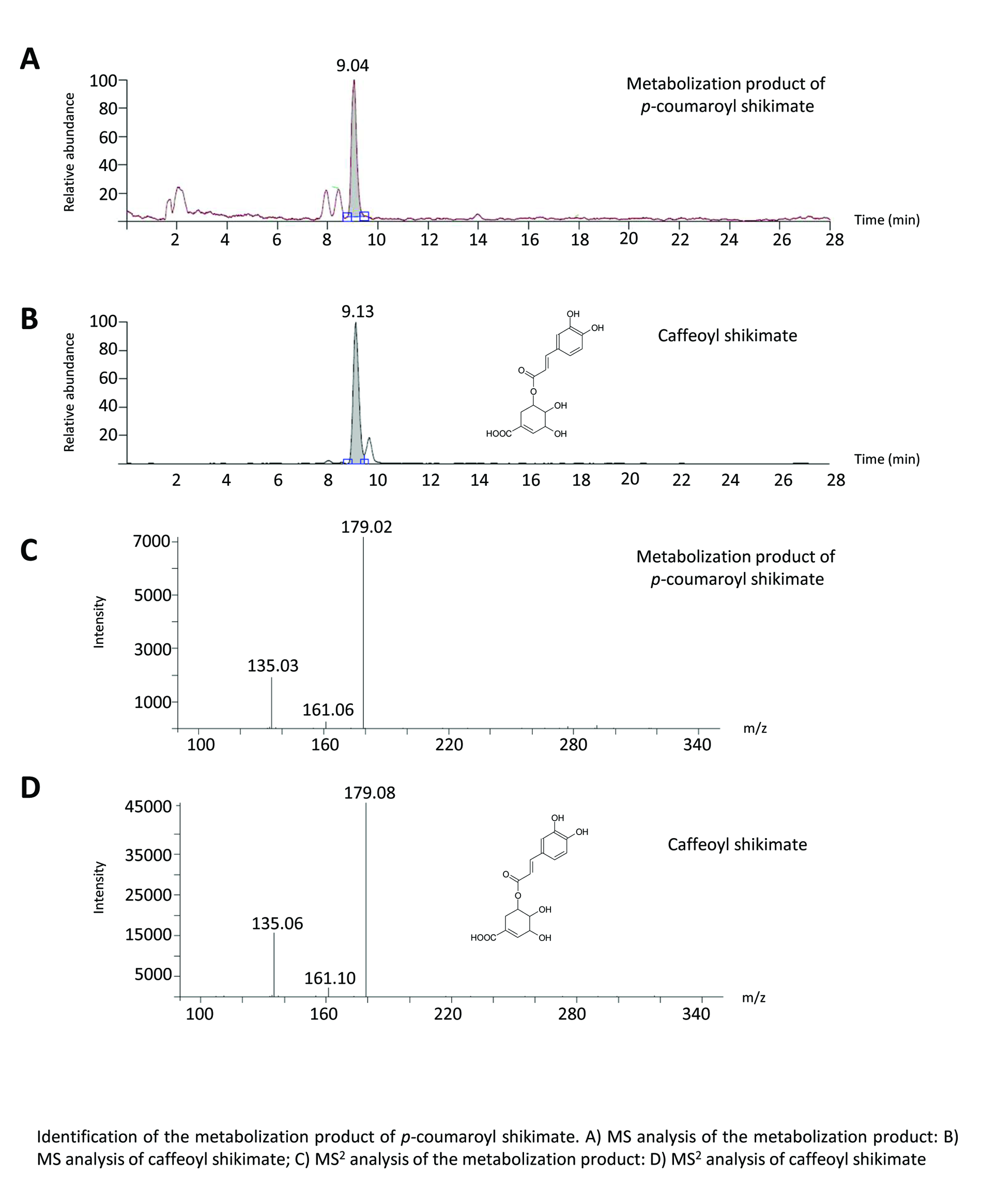

Supplement: Additional file 3 — Identification of the metabolization product of p-coumaroyl shikimate. A) MS analysis of the metabolization product: B) MS analysis of caffeoyl shikimate; C) MS2 analysis of the metabolization product: D) MS2 analysis of caffeoyl shikimate. [file 1471-2229-12-152-S3.tiff]

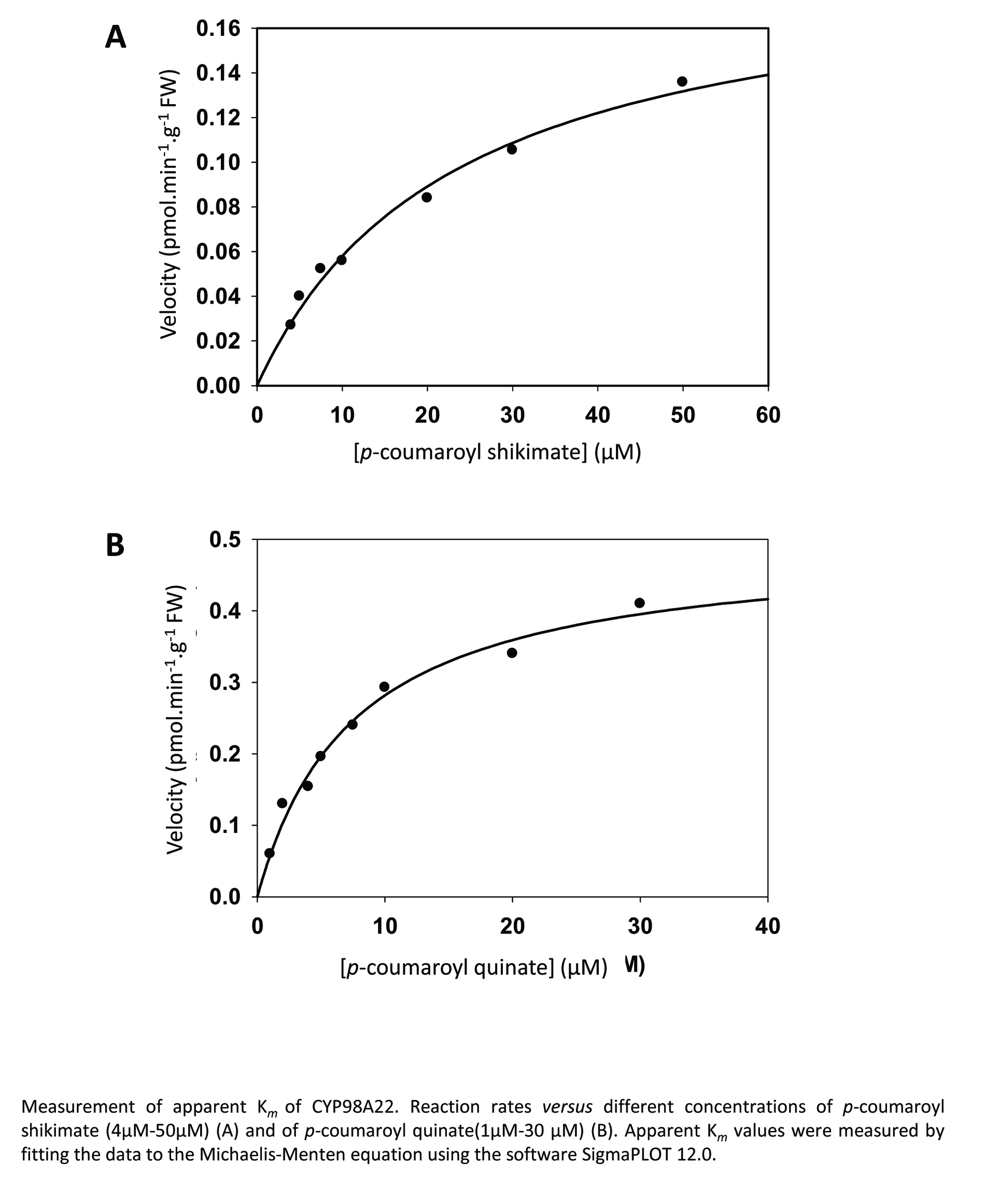

Supplement: Additional file 4 — Measurement of apparent Kmof CYP98A22. Reaction rates versus different concentrations of p-coumaroyl shikimate (4μM-50μM) (A) and of p-coumaroyl quinate(1μM-30 μM) (B). Apparent Km values were measured by fitting the data to the Michaelis-Menten equation using the software SigmaPLOT 12.0. [file 1471-2229-12-152-S4.tiff]
